# Platelet-Derived Growth Factor-Functionalized Scaffolds for the Recruitment of Synovial Mesenchymal Stem Cells for Osteochondral Repair

**DOI:** 10.1155/2022/2190447

**Published:** 2022-01-27

**Authors:** Yuan Luo, Xiaodong Cao, Junfeng Chen, Jianwei Gu, Hao Yu, Junying Sun, Jun Zou

**Affiliations:** ^1^Department of Orthopedic, The First Affiliated Hospital of Soochow University, Suzhou, Jiangsu, China; ^2^Department of Orthopedic, Taicang Affiliated Hospital of Soochow University, Taicang, Jiangsu, China

## Abstract

Cartilage regeneration is still a challenge for clinicians because of avascularity, denervation, load-bearing, synovial movement, and the paucity of endogenous repair cells. We constructed a multilayered osteochondral bionic scaffold and examined its repair capacity using a rabbit osteochondral defect model. The cartilage phase and interface layer of the scaffold were prepared by freeze-drying, whereas the bone phase of the scaffold was prepared by high-temperature sintering. The three-phase osteochondral bionic scaffold was formed by joining the hydroxyapatite (HAp) and silk fibroin (SF) scaffolds using the repeated freeze-thaw method. Different groups of scaffolds were implanted into the rabbit osteochondral defect model, and their repair capacities were assessed using imaging and histological analyses. The cartilage phase and the interface layer of the scaffold had a pore size of 110.13 ± 29.38 and 96.53 ± 33.72 *μ*m, respectively. All generated scaffolds exhibited a honeycomb porous structure. The polydopamine- (PDA-) modified scaffold released platelet-derived growth factor (PDGF) for 4 weeks continuously, reaching a cumulative release of 71.74 ± 5.38%. Synovial mesenchymal stem cells (SMSCs) adhered well to all scaffolds, but demonstrated the strongest proliferation ability in the HSPP (HAp-Silk-PDA-PDGF) group. Following scaffold-induced chondrogenic differentiation, SMSCs produced much chondrocyte extracellular matrix (ECM). In *in vivo* experiments, the HSPP group exhibited a significantly higher gross tissue morphology score and achieved cartilage regeneration at an earlier stage and a significantly better repair process compared with the other groups (*P* < 0.05). Histological analysis revealed that the new cartilage tissue in the experimental group had a better shape and almost filled the defect area, whereas the scaffold was nearly completely degraded. The new cartilage was effectively fused with the surrounding normal cartilage, and a substantial amount of chondrocyte ECM was formed. The SF/HAp three-layer osteochondral bionic scaffold exhibited favorable pore size, porosity, and drug sustained-release properties. It demonstrated good biocompatibility in vitro and encouraging repair effect at osteochondral defect site in vivo, thereby expected to enabling the repair and regeneration of osteochondral damage.

## 1. Introduction

Due to low cellularity and lack of vascularity and innervation, damaged articular hyaline cartilage often cannot repair itself spontaneously [1] and is prone to further degeneration when subjected to exercise and load, resulting in pain and dysfunction [2]. Damage to the articular cartilage can be divided into two types: partial-thickness damage and full-thickness damage. Among the most challenging aspects of clinical practice is the repair of full-thickness cartilage damage [3]. All available clinical treatment methods present problems, such as secondary injury and donor limitations, as well as difficulty in achieving long-term stable curative effects due to the inability to regenerate hyaline cartilage [4, 5]. Articular cartilage, subchondral bone, and bone together form a complete functional unit, exhibiting anatomical gradient and physiological specificity, while being closely connected in anatomical structure. In particular, the biomechanical properties, metabolic characteristics, and material transport capabilities vary from one region to another. Therefore, research on bionic tissue engineering scaffolds has advanced significantly in recent years. Besides providing a frame for seed cells to adhere, proliferate, and differentiate, scaffolds can also serve as a carrier for the release of growth factors. In addition, they can also offer a mechanically stable environment to facilitate tissue regeneration [6–8]. SF is a natural polymer material, ideal for use as a scaffold in tissue engineering. The structure of SF is similar to that of collagen in normal tissues. Concomitantly, it also possesses favorable biocompatibility, mechanical properties, and a certain degree of biodegradability, making it a hot spot in regeneration research [9]. HAp is a calcium phosphate mineral that has gained widespread attention because of the similarity of its chemical composition and crystal structure to those of natural bone minerals. It has been successfully used in clinical bone repair and has produced satisfactory curative results [10].

The ability to differentiate into cartilage is the primary criteria used by researchers for the selection of seed cells and their reliable source, which is also an important factor for consideration. Recent studies have shown that SMSCs can efficiently proliferate and differentiate into cartilage and exist in a large number in the synovium around the joint, which makes it easy to obtain [3, 11]. In tissue engineering, growth factors are critical regulatory factors that control the proliferation rate, migration direction, and differentiation potential of cells. PDGF is a basic protein stored in platelet *α* particles. With cartilage injury, the coagulation mechanism helps to form many blood clots at the injury site, which contain platelets rich in PDGF [12]. However, macrophages that have migrated due to inflammation also synthesize large amounts of PDGF to promote the proliferation and differentiation of remaining chondrocytes nearby, achieving the local regeneration and repair of cartilage [13].

Many previous studies need to culture cells on scaffolds in advance, which has been damaged by multiple operations. The cell-free scaffold in this study can be implanted through one operation to avoid two-stage injuries and to recruit stem cells at the injured site for in situ repair. The structure, porosity, and pore size range of the scaffold material were examined under a scanning electron microscope (SEM). PDA was used to increase the active groups on the surface of the material, increasing the loading of PDGF to the scaffold material and achieving sustained release. The biological performance of cells on the scaffold material was also examined. Furthermore, scaffolds were implanted in rabbit osteochondral defect models, and the effects of osteochondral damage repair were assessed using imaging and histological indicators. Our study indicated that a cell-free tissue engineering scaffold can be constructed and growth factors within the scaffold can recruit SMSCs around the cartilage damage site to facilitate cartilage repair.

## 2. Materials and Methods

### 2.1. Construction and Characterization of the SF/HAp Three-Phase Scaffold [14]

#### 2.1.1. Construction

**Figure 1:**
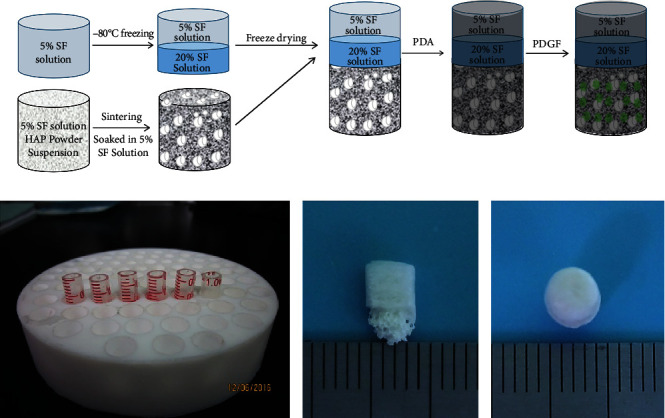
(a) Schematic illustration of the preparation of the three-phase scaffold. (b) Custom mold for making scaffold. The profile (c) and vertical (d) appearance of scaffold.

SF solutions from cocoons of *B. mori* (RudongXinsilu Co., Ltd., Jiangsu, China) were prepared using the following methods. SF was added to 0.5% Na_2_CO_3_ solution, boiled for 1 h, and dissolved in 9.3 mol/L lithium bromide solution (Strem Chemicals Inc., MA, USA). Repeated dialysis with ultrapure water and concentrating with ethylene glycol were performed to prepare SF solutions of different concentrations. HAp powder (Aladdin, Shanghai, China) was added to the 5% SF solution and sintered for 3 h; it was injected into a mold with a diameter of 4 mm and a length of 10 mm, placed in a refrigerator at -80°C overnight, and freeze-dried to obtain a scaffold. The scaffold was placed in a muffle furnace, and the temperature was raised to 90°C at 1.5°C/min, then to 200°C for 40 min, after it was risen further to 1250°C at 2°C/min, and sintered at this temperature for 4 h. The 5% SF solution was added to the mold and then placed in the refrigerator at -20°C overnight. The 20% SF solution was preheated to 45°C and dropped into the previous mold, which was then placed in the refrigerator at -80°C overnight. Subsequently, the HAp scaffold sample soaked in SF solution was preheated to 45°C and placed into the mold, then freezing overnight at -80°C. The SF/HAp three-layer osteochondral bionic scaffold was obtained by freeze-drying (Alpha 1e2 LD plus, Christ, Germany). After soaking in methanol for 30 min, the structure of SF was altered and an insoluble SF/HAp three-layer osteochondral bionic scaffold was obtained as illustrated in Figure 1.

#### 2.1.2. SEM Observation of Scaffold Structure

The scaffold samples were cut to 2–3 mm thickness and adhered to the conductive adhesive on the sample stage. SEM samples were then sprayed with gold in the ion sputter coater for observation. Analysis of the pore size of the scaffold was performed using NanoMeasurer. Scaffold porosity was calculated using the liquid replacement method.

### 2.2. Load and Release of PDGF on the Scaffold

The above bionic scaffold was immersed in 2 mg/mL dopamine solution and shaken on a shaker at 20°C for 24 h. The scaffold was then ultrasonically cleaned with deionized (DI) water and dried overnight in a vacuum. The scaffold was sterilized by ultraviolet (*λ* = 254 nm) irradiation for 4 h before use. In the experimental design of this study, PDGF (Peprotech, USA) was only loaded on the cartilage-phase SF scaffold. Therefore, only the pure SF scaffold group (SP) and PDA-modified SF scaffold group (SP-PDA) were considered in this part of the study. *In vitro* PDGF release curves were measured using ELISA.

### 2.3. Evaluation of Biological Behaviors of Cells on the Scaffold

#### 2.3.1. Cell Proliferation

The scaffolds were first placed in 96-well plates according to groups. After irradiation sterilization, a 1 mL cell suspension (total of 2 × 10^3^ cells) was dripped onto the scaffold and cultured in a 5% CO_2_ incubator at 37°C with complete medium. The proliferation rate of cells in each group at different time points (1, 3, 5, and 7 d) was measured using the CCK-8 method, and the corresponding proliferation curves were plotted.

#### 2.3.2. Cell Migration

A 24-well Transwell plate was selected, 150 *μ*L cell suspension was added to the upper chamber, and 700 *μ*L serum-free medium was added to the lower chamber. After 24 h of starvation, according to the added component, the lower chambers were set as the pure SF scaffold group (SF), PDA-modified SF scaffold group (SP-2), PDA-modified and PDGF-loaded SF scaffold group (SPP), 50 ng/mL PDGF-supplemented DMEM group (DMEM+PDGF), and 5% FBS-supplemented DMEM group (DMEM+FBS). After overnight incubation, the upper chamber was taken out and the inner surface was scraped several times using cotton swabs. Only those cells that passed through the upper chamber and attached to the outer side of the membrane were retained. Cells were counted under a fluorescence microscope after staining with crystal violet.

#### 2.3.3. Pellet Culture

The pellet culture was used to induce the differentiation of SMSCs into cartilage. P3 SMSCs were adjusted to 1 × 10^5^/mL and 1 mL cell suspension taken for centrifugation to obtain SMSC agglomerates. Care was taken over the process to avoid blowing the agglomerates; they were gently transferred to an ultrafiltration centrifuge tube, and different group bionic scaffolds were placed in the inner chamber for pellet induction culture. The diameters of pellets in different groups were measured. After paraffin embedding, alcian blue, and toluidine blue staining were applied to analyze the specific expression of ECM in pellets. In this part of the study, only the SP-2 group (PDA-modified SF scaffold) and the SPP group (PDA-modified and PDGF-loaded SF scaffold) were considered.

#### 2.3.4. Cytoskeleton Staining

A Transwell culture was employed to induce cartilage formation, and the cytoskeleton and nuclei of cells were immunofluorescence stained to visualize the expression and arrangement of cytoskeleton proteins in each group.

### 2.4. Construction of the Rabbit Osteochondral Defect Model and Implantation of the Three-Phase Scaffold

New Zealand white rabbits were purchased from the Experimental Animal Center of Soochow University (Suzhou, China). The animal handling and surgical procedures were conducted in accordance with protocols approved by the Ethics Committee at the First Affiliated Hospital of Soochow University. A 4 mm diameter trephine was used to construct an osteochondral defect (4 mm in diameter and 5 mm in depth) at the trochlear part of the rabbit knee joint, where a scaffold was implanted. The following groups were set up: normal, blank, HSF, HSP-1, HSP-2, and HSPP as listed in Table 1.

#### 2.4.1. MRI

We conducted magnetic resonance imaging (MRI) examinations at 6, 12, and 24 weeks after surgery. We used the 1.5 T GE Signa magnetic resonance imager (Signa HDe; GE) to perform a routine scan of the sagittal T2-weighted image (Sag 3D SPGR fat sat sequence) of rabbit's knee joint and scored according to the MOCART MRI evaluation standard.

#### 2.4.2. Staining

Samples were harvested according to the set time points and evaluated based on International Cartilage Repair Society (ICRS) scores. After serial treatment, HE, Masson, and Safranin O/fast green staining, as well as immunohistochemical staining of aggrecan, Col-I, and Col-II, were conducted on the sections to analyze the cartilage regeneration status and specific ECM expressions.

### 2.5. Statistics

All samples were tested in triplicates (unless indicated), and values were expressed as the mean (M) ± standard deviation (SD). GraphPad Prism (GraphPad Software, Inc.; USA) were used for statistical analysis and plotting. Biohistochemical analysis and gene expression analysis were performed using one-way ANOVA and Tukey's test. Pairwise comparison between groups was performed using an independent sample *t*-test. *P* < 0.05 indicated a statistically significant difference between groups.

## 3. Results and Discussion

### 3.1. Preparation and Characterization of the Osteochondral Bionic Scaffold

In consideration of the tissue sections of normal rabbit knee joint and the requirements of tissue engineering scaffolds suitable for different cell growth, we selected the concentration of SF according to the pore size distribution and porosity under electron microscope. We finally selected the 5% SF solution for the cartilage phase, whereas the 20% SF solution was chosen as the working concentration for the interface layer. The constructed scaffold was approximately 4 mm in diameter and 5 mm in height, consistent with the dimensions of the critical defect reported in the literature [15].

**Figure 2:**
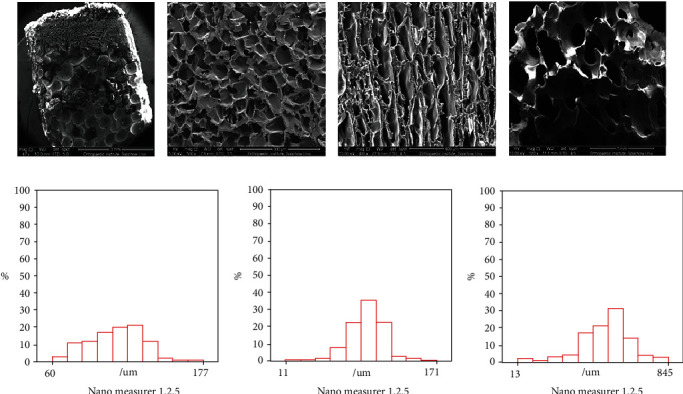
SEM image of porous structure of the scaffold ((a) scale bar = 3 mm) and the gradual porous structure of cartilage phase ((b) scale bar = 300 *μ*m), interface layer ((c) scale bar = 400 *μ*m), and bone phase ((d) scale bar = 1 mm). Pore size distribution of cartilage phase (e), interface layer (f), and bone phase (g) under nanomeasure.

The scaffold, which was cylindrical, was divided into three layers along its long axis. The height of cartilage layer was 1 mm, the interface layer 1 mm, and the subchondral bone, and bone phase was about 3 mm as shown in Figures 1(c) and 1(d). The pore size of the cartilage phase was in range of 60–177 *μ*m, the interface layer 27–171 *μ*m, and the bone phase 96–845 *μ*m as shown in Figure 2. Interestingly, the pore size distribution of each layer was generally uniform. However, pore sizes within the cartilage phase, the interface layer, and the bone phase were not consistent, closely mimicking the normal anatomical structure. We also found that the porosity of each experimental group was not significantly changed compared to that of the blank group (*P* > 0.05), which was maintained at approximately 45%.

### 3.2. Load and Release of PDGF

**Figure 3:**
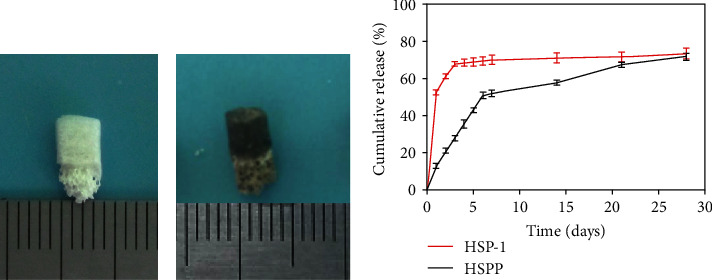
The appearance of scaffold treated (a) without and (b) with polydopamine and (c) *in vitro* release profiles of PDGF from HSP-1 and HSPP scaffold.

Following the PDA modification of the scaffold, a uniform coating developed on its surface, resulting in a gray-black appearance like Figures 3(a) and 3(b). According to the minimum effective concentration, 50 ng PDGF was diluted into 100 *μ*L solution and then dropped onto the physical adsorption group and PDA group. In this experiment, we only loaded PDGF on the SF scaffold of the cartilage phase; thus, only the HSP-1 and HSPP groups were investigated.

Because of the physical adsorption of PDGF, we observed a 52.5% burst release in the HSP-1 group on the first day. On the third day, the release reached 67.74%, whereas on the 28th day, the cumulative release reached approximately 73.41%. In comparison, we observed no burst release of PDGF in the HSPP group on the first day; instead, in this study, only 12.53% PDGF was released. However, on the seventh day, the gradual release reached 52.07%, whereas on the 28th day, the cumulative release reached 71.74% as shown in Figure 3(c).

### 3.3. Evaluation of the Biological Behaviors of Cells on the Scaffold

#### 3.3.1. Cell Adhesion and Proliferation Experiments

**Figure 4:**
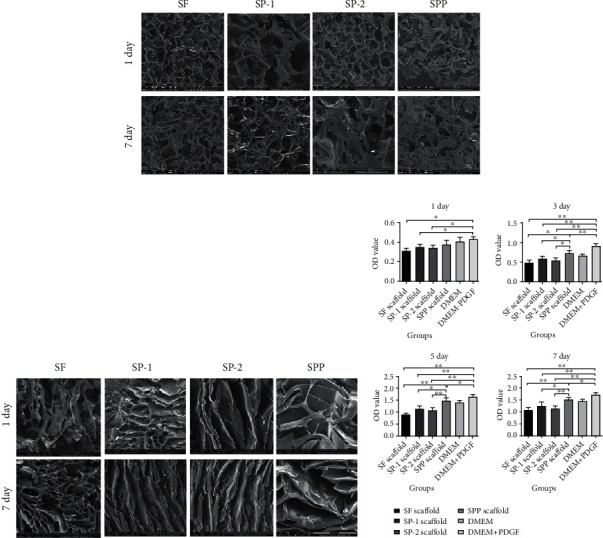
Cell adhesion and proliferation assays of different scaffolds. (a) SEM images of SMSCs adhesion on cartilage phase of scaffold loaded with or without PDGF or PDA after seeding for 1 d and 7 d. (b) SEM images of SMSC adhesion on interface layer of scaffold after seeding for 1 d and 7 d. (c) Cell proliferation of SMSCs cultured on different scaffolds after 1 d, 3 d, 5 d, and 7 d using a CCK-8 kit. Statistically significant differences are indicated with ^∗^*P* < 0.05 and ^∗∗^*P* < 0.01 compared with control.

For this experiment, we seeded P3 SMSCs onto each group of scaffolds and observed cells under a SEM microscope at the set time points. We observed that the PDGF-loaded SP-1, SPP, and DMEM+PDGF groups all demonstrated an obvious increase in the total content of cells, suggesting that PDGF had a significant promoting effect on cell proliferation (*P* < 0.05). Among all groups, the SPP group exhibited the most significant increase in the number of cells as shown in Figure 4(*P* < 0.05).

#### 3.3.2. Migration Experiment

**Figure 5:**
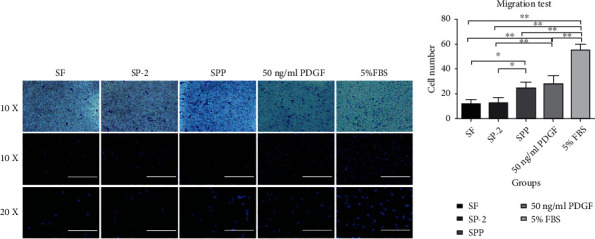
Transwell migration assay of the effects of PDGF released from the scaffolds on SMSCs migration. (a) Microscopic images of crystal violet and fluorescent staining of cells cultured in media that contained control or scaffold loaded with PDGF after 24 h of culture. (b) Quantitative comparison of migrated cells among the different groups, ^∗^*P* < 0.05 and ^∗∗^*P* < 0.01 compared with control.

We used Transwell culture plates in this experiment. We placed all scaffold samples in the lower chamber and seeded SMSCs in the upper chamber. After 24 h of coculture, cells adhering to the outside after penetrating the membrane were visualized using crystal violet and fluorescence staining. Five visual fields were selected for cell counting and plotting. The results in Figure 5 demonstrated that 5% FBS showed the strongest migration promoting effect (*P* < 0.01), and the simple DMEM + PDGF group was second only to the serum group, but there was no significant difference compared with the SPP group, indicating that the PDGF released by the scaffold could reach the effective working concentration of 50 ng/mL. There was no significant difference between SF group and SP-2 group (*P* < 0.05), indicating that PDA could not promote the migration of SMSCs.

#### 3.3.3. Chondrogenic Differentiation

**Figure 6:**
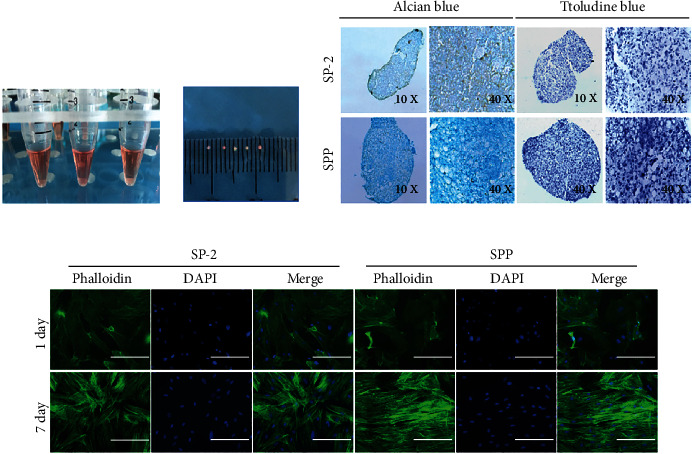
(a) Schematic diagram of pellet culture and (b) general view of pellet. (c) Alcian blue and toluidine blue staining showed the secretion of cartilage-specific extracellular matrix. (d) After 7 d coculture of SMSCs and scaffolds, the cytoskeleton was stained with phalloidin, and the nucleus was stained with DAPI to show the arrangement of skeletal proteins. Scale bar = 200 *μ*m.

We performed the pellet culture experiment using the SP-2 and SPP groups to compare the differentiation-promoting effects of PDGF. After 14 d of culture, we embedded the scaffold samples in paraffin and stained the sections using alcian blue and toluidine blue. We observed that the pellet volume of the SPP group was slightly larger than the SP-2 group, and pellets were wrapped in a large amount of transparent ECM as shown in Figures 6(a) and 6(b). Observations of sections in Figure 6(c) revealed the SPP group displayed significantly better staining than the SP-2 group (*P* < 0.05), indicating that pellets contained large amounts of proteoglycan, hyaluronic acid, and epithelial acid mucin.

#### 3.3.4. Cytoskeleton Staining

We also used Transwell plates for cell culture. We placed the scaffolds of each group in the upper chamber, whereas cells were seeded in the lower chamber. After 7 d, the number of cells in the SPP group was significantly higher than that in the SP-2 group (*P* <0.05), further demonstrating the promoting effect of PDGF on cell proliferation. By staining the cytoskeleton, furthermore, it changed from a short and wide shape into a long fusiform one. We further noticed that cells in the SPP group exhibited more orientation, and their skeleton proteins followed a more regular arrangement, with a tendency to fuse as shown in Figure 6(d). This finding was like the orderly arrangement of ECM in the superficial layer of cartilage, such as Col-II.

### 3.4. Role of the Osteochondral Three-Layer Bionic Scaffold in Cartilage Repair in the Rabbit Osteochondral Defect Model

**Figure 7:**
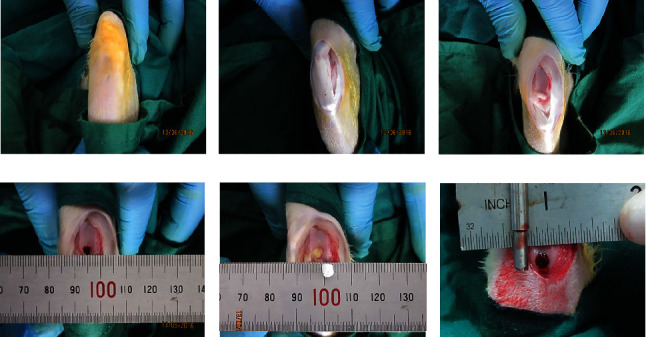
Modeling osteochondral defect and scaffold implantation. (a) Skin preparation after routine anesthesia. (b) Medial patellar incision. (c) Exposure of femoral trochlea. (d) Femoral trochlea osteochondral defect was successfully modeled. Three-phase scaffold (e) without and (f) with PDA treatment was implanted.

To evaluate the effect of the scaffolds loaded with PDGF, as grafts to facilitate cartilage formation *in vivo*, we surgically created an osteochondral defect of 4 mm diameter and 5 mm depth in their femoral condyle using a trephine. Subsequently, we implanted scaffolds of the groups in the defect area as shown in Figure 7.

#### 3.4.1. MRI Examinations of Scaffold Samples

**Figure 8:**
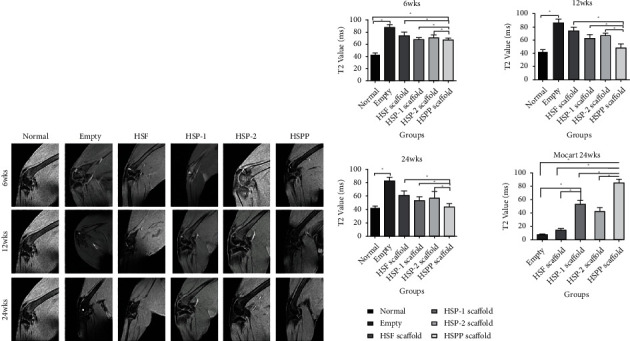
(a) MRI images of osteochondral defect regeneration in different groups at set time points and (b) their T2 value and MOCART scores of each group at 24 weeks.

The sagittal scan of the T2 phase allows for a more intuitive visualization of the defect site, facilitating a better analysis of the reconstruction of the osteochondral tissue. By analyzing the MRI results, high signals were detected in the osteochondral defect area in the blank group at 6, 12, or 24 weeks, suggesting a lack of collagen filling in the local defect area. In contrast, water-like fillings were observed in the T2 phase. In comparison, the rest of the groups showed varying degrees of repair, with the surface producing cartilage-like tissues locally, including but not limited to fibrocartilage and hyaline cartilage as shown in Figure 8(a). We also noticed that the HSPP group had almost no high signal at 12 weeks, indicating the successful local tissue repair. We further observed that the 24-week MRI image was like that of a normal knee joint, suggesting that the regeneration and repair tissue in the HSPP group was comparable to normal articular cartilage. Interestingly, in this study, the relaxation time was also significantly shorter than other experimental groups as shown in Figure 8(b) (*P* < 0.05).

#### 3.4.2. Gross Morphology Score of Neocartilage

**Figure 9:**
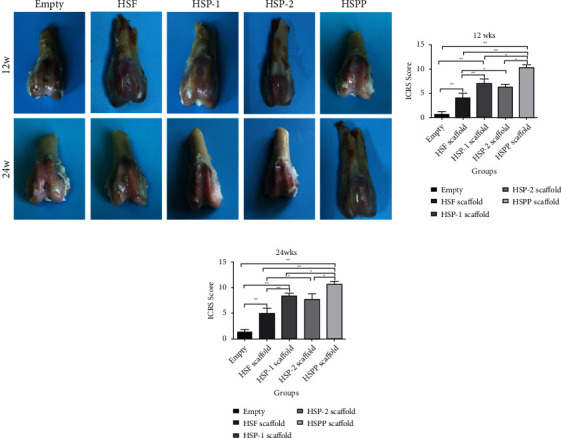
Appearance of gross specimens and ICRs scores at 12 and 24 weeks in each group.

We scored the gross morphology of the formed neocartilage according to the Visual Histological Assessment Scale published by the ICRS. The scaffold treatment groups performed significantly better than the blank group, whether at 12 or 24 weeks as shown in Figure 9(a) (*P* < 0.01). These findings demonstrated that, when osteochondral damage occurs, corresponding interventions are required, which can effectively improve the degree of osteochondral repair. In this study, even the score of the blank scaffold group was significantly higher than the blank group as shown in Figure 9(b) (*P* < 0.05), indicating that the filling of the porous scaffold provided a favorable growth environment for cells. Furthermore, the HSPP group exhibited the best cartilage repair effect as more PDGF was released early to recruit surrounding SMSCs to the osteochondral bionic scaffold.

#### 3.4.3. Staining of Scaffold Samples

**Figure 10:**
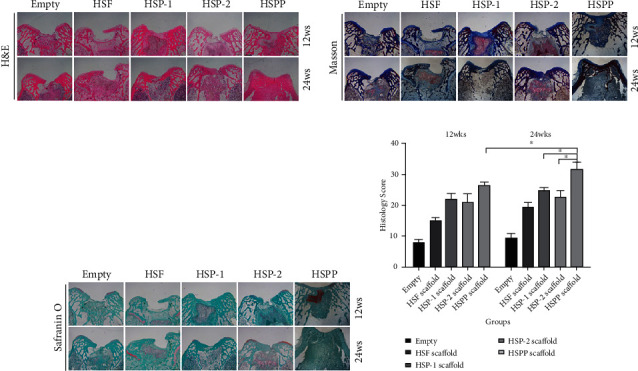
Histological evaluation of cartilage repair with different scaffolds in osteochondral defect models. (a) H&E staining. (b) Masson staining. (c) Safranine O fast green staining. (d) Histological scores at 12 and 24 weeks in each group.

**Figure 11:**
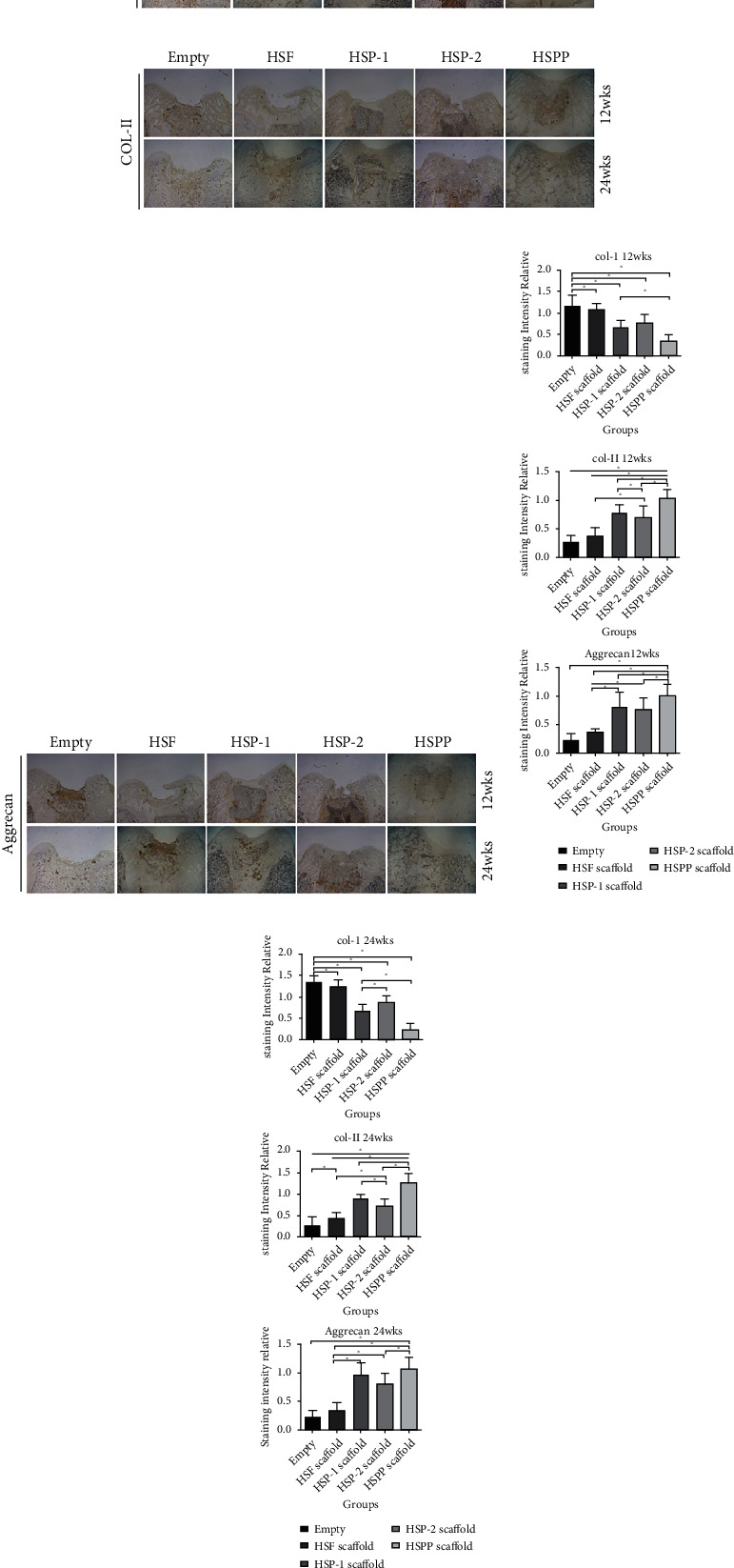
Immunohistochemical results of repaired tissues with different scaffolds. (a) CoL-I immunohistochemical staining. (b) Col-II immunohistochemical staining. (c) Aggrecan immunohistochemical staining. Relative expression of CoL-I, Col-II, and Aggrecan in different groups at (d) 12 weeks and (e) 24 weeks.

Samples were harvested at 12 and 24 weeks. Following serial treatment, we performed H&E, Masson, and Safranin O/fast green staining in Figure 10, as well as immunohistochemical staining of Col-I, Col-II, and aggrecan in Figure 11. Using these different staining methods, we examined the regeneration of cartilage, secretion of ECM, and expression of cartilage-specific proteins.

We observed that large cavities and defects were still visible in the blank samples after staining. These cavities were filled by some fibrous cells, covering less than 50% of the area. We also found that the subchondral bone was partially filled with compact bone and fibrous tissue, resulting in loss of the basic structure of normal chondrocytes. However, we observed no cartilage lacuna around cells, and relatively little ECM was present. Our immunohistochemical analysis suggested the formation of significant Col-I in the repair tissue, whereas both cartilage-specific Col-II and aggrecan were absent (*P* < 0.05).

In the HSF group, our staining results indicated substantial filling defects in the samples. The defect area was filled with a significant amount of newly formed fibrous tissue (*P* < 0.05), which was slightly thicker than the surrounding normal cartilage. Only a few surface cracks were visible on the surface, and Safranin O staining was negative. In contrast, we observed little positive staining at the junction between the new tissue and the surrounding normal cartilage, suggesting a tendency for the formation of new cartilage at the junction. Our immunohistochemistry results indicated that the repaired tissues were mainly composed of Col-I, which exhibits relatively high strength, whereas lacking the expressions of cartilage-specific Col-II and aggrecan.

In both the HSP-1 and HSP-2 groups, repaired tissues accounted for over 50%, with over 50% of the scaffold material being degraded. We also observed a bilateral partial fusion in the repaired tissues around the normal cartilage, whereas the defect area was filled with hyaline cartilage-like repaired tissues. In addition, Safranin O staining was low positive near the cartilage area, and lacuna-like structures were evident in regenerated tissues. Although cells were scattered, there were still many fibroblasts present in the central region of the repaired tissue. According to our immunohistochemistry results, the repaired tissues exhibited expression of Col-I, Col-II, and aggrecan.

We further observed an excellent performance of cartilage repair in the HSPP group, exhibit a significant amount of specific ECM formation (*P* < 0.05). Afterwards, under the continuous action of PDGF, SMSCs differentiated into cartilage, secreting significant specific ECM (*P* < 0.05). At 12 weeks, we observed the perfect filling of new tissues, whereas the scaffold had been degraded by over 50%. Moreover, the new cartilage, which was a smooth hyaline cartilage-like tissue, was similar in thickness to the surrounding cartilage and it. As of 24 weeks, we detected the defect was almost filled with new cartilage, and the scaffold was almost completely degraded. The subchondral bone was normal cancellous bone, whereas the new cartilage was normal hyaline cartilage, which was well fused with surrounding normal cartilage. Safranin O staining was positive and immunohistochemistry suggested the high expression of Col-II and aggrecan in the repaired tissues. We detected expression of Col-I only in the interface layer. These findings indicated SMSCs differentiated well into cartilage, forming considerable cartilage-specific ECM.

## 4. Discussion

Due to its unique anatomical structure and the absence of nerves, blood vessels, and lymphatic system, articular hyaline cartilage receives most of its nutrition from the synovial fluid. Therefore, the articular cartilage has a limited capacity to repair itself once damaged [1, 3]. Because of the unique cartilage anatomy, researchers learned of the concept of osteochondral units and designed scaffolds with multilayer structures for cartilage repair [6–8]. According to Hunziker, growth factors are effective in promoting the migration of SMSCs to partially damaged cartilage for repair [16]. If full-thickness occurs osteochondral damage, the osteochondral units should be repaired. Xu et al. demonstrated the efficient repair of full-thickness osteochondral damage and evaluated gross and histological scores. They found that in the experimental group exhibiting good repair effects, both the subchondral bone and bone were well reconstructed, further confirming the definition of the osteochondral unit [3]. In this study, an SF/HAp three-layer osteochondral bionic scaffold was constructed successfully. The scaffold surface was modified with PDA and then loaded with PDGF. The surrounding endogenous pluripotent stem cells, SMSCs, were recruited to the damage site and induced to differentiate into cartilage, achieving a one-step tissue engineering repair of osteochondral damage.

The SF/HAp three-layer osteochondral bionic scaffold constructed in this study exhibited a sponge-like porous structure with a porosity above 40%. The pore size distributions of the three layers were generally uniform; however, the pore sizes were inconsistent among the three layers. The pore size of the cartilage phase was mainly in the 100–130 *μ*m range. According to previous studies, this diameter is more favorable for the growth of chondrocytes, providing an ideal environment for cell adhesion. An appropriate pore size is also conducive to the differentiation of cells into cartilage [17, 18]. The pore size of the interface layer was mainly in the 75–100 *μ*m range, which is relatively small and thus not conducive to the growth and passage of cells. To a certain extent, this layer could block the circulation of the medullary cavity and bone marrow cavity cells, as well as the transport of some nutrient molecules and metabolites [1]. Finally, the pore size of the bone phase was mainly in the 350–600 *μ*m range. It has been previously reported that a pore size within this range is conducive to the adhesion and differentiation of osteoblasts, and the osteogenic ability of porous HAp scaffolds has also been widely confirmed in many studies [19, 20].

To achieve the loading and sustained release of PDGF, PDA was employed in this study to modify the scaffold surface. As dopamine contains a large amount of phenolic hydroxyl and amino groups, it serves as a good platform and can thus be used for grafting reactions [21, 22]. Wang et al. confirmed that, by immobilizing the nanocomposite on the surface of the self-polymerizing coating of PDA, both the adhesion and spread of stem cells were effectively promoted, and the secretion of ECM was further enhanced [23]. *In vitro* release experiments performed in this study demonstrated that the PDA-modified osteochondral bionic scaffold prevented the burst release, allowing PDGF to be continuously released for 3 weeks, reaching a cumulative release of 71.74%. Thus, we demonstrated the effective loading and sustained release of growth factors in the scaffold, which promoted the differentiation of SMSCs toward cartilage and the synthesis of ECM.

Stem cell mobilization is widely regarded as a promising approach to treat orthopedic diseases, especially when using endogenous stem cells, which are more readily available [4, 24, 25]. The synovium is a loose connective tissue in the inner layer of the articular capsule. The embryonic origin of this tissue is like that of cartilage, as is its expression of surface antigens and differentiation potential [26]. If cartilage damage occurs, regeneration and repair signals are generated; these signals trigger the migration of SMSCs to the defect site to complete the repair process [13]. In addition, SMSCs have a rapid proliferation rate and a controlled differentiation ability, leading them to become one of the most popular seed cells in the research of tissue engineering for cartilage repair [27]. PDGF is a basic protein stored in platelet *α* particles. When osteochondral damage occurs, the local damage site develops many blood clots, which are rich in PDGF. Concurrently, macrophages that migrate in response to local damage synthesize a large amount of PDGF to promote the proliferation and differentiation of the remaining nearby chondrocytes, completing local cartilage regeneration and repair [12, 28, 29]. The *α* and *β* receptors present on the surface of SMSCs allow PDGF-AB to exert a strong recruitment effect on them. Further, locally produced PDGF induces SMSCs in the peripheral synovium to migrate to the damaged area and proliferate [12]. Many studies have also demonstrated that under the action of PDGF, SMSCs differentiate into cartilage, secrete a large quantity of chondrocyte ECM arranged horizontally similar to the superficial layer of cartilage, maintain chondrocyte phenotypes, and complete osteochondral repair [13]. The arrangement of the generated ECM exhibits the advantages of locking moisture, improving tensile strength, distributing joint load, and facilitating joint movement. Thus, it affects the formation, growth, repair, and regeneration of cartilage [30].

The ultimate evaluation criterion is the *in vivo* cartilage repair effect. In this study, our MRI results showed that the blank group lacked collagen filling in the defect area, whereas the other groups showed varying degrees of repair. The HSPP group showed almost no high signals in the defect area at 12 weeks. At 24 weeks, its MRI image was almost identical to that of the normal knee joint, most notably in terms of cartilage defect filling, the fusion of the repaired tissue with the surrounding normal cartilage, the cartilage signal strength, and the internal structure of the repaired tissue. The HSPP group scored significantly higher than the other experimental groups (*P* < 0.05), indicating its promising cartilage repair effect. Trattnig et al. also used the T2 quantitative map to evaluate the articular cartilage repair effect, which can indicate the cartilage function and a more accurate assessment of the prognosis [31]. Observations of postoperative gross samples with blood scabs and inflammation during local damage revealed that macrophages could synthesize and secrete PDGF. However, because of the low concentration, early recruitment of surrounding SMSCs can be difficult. Instead, the nearby fibroblasts and bone marrow mesenchymal stem cells (BMSCs) in the medullary cavity will occupy the damaged site preemptively. Therefore, when osteochondral damage exceeds a critical defect, it is difficult for the damage to heal on its own, and much fibrous tissue is formed. A 3D porous scaffold with an oriented structure constructed by Liu et al. was effective at repairing osteochondral defects, further highlighting the significance of scaffold filling [32]. At 24 weeks, no significant difference was observed between the repaired and surrounding normal cartilage in the HSPP group (*P* < 0.05). Both tissues exhibited a red hyaline cartilage-like structure, exhibiting good luster and elasticity, showing no obvious separation boundaries, and demonstrating a good ability for repair and regeneration. Histological analysis showed that the relatively early release of high concentrations of PDGF led to the recruitment of many SMSCs to the damaged site, where they were differentiated into cartilage, secreting significant specific ECM under the continuous stimulation by PDGF (*P* < 0.05). Therefore, the HSPP group demonstrated perfect new tissue filling at 12 weeks, whereas at 24 weeks, the defect area was almost filled by the new cartilage, and the scaffold was nearly completely degraded. The formed subchondral bone was a normal cancellous bone, whereas the new cartilage, which was hyaline-like, fused well with the surrounding normal cartilage, exhibiting a positive Safranin O staining. Finally, immunohistochemical analysis indicated the high expression of Col-II and aggrecan in the repaired tissues and the formation of substantial cartilage-specific ECM.

## 5. Conclusions

The three-layer osteochondral bionic scaffold composed of SF and HAp met the requirements of osteochondral tissue engineering. Treatment of the scaffold with PDA allowed the loading and sustainable release of growth factors. In addition, the scaffold exhibited high biocompatibility and promoted the adhesion, proliferation, migration, and differentiation of SMSCs into cartilage. *In vivo* experiments showed that the three-layer SF/HAp osteochondral scaffold effectively filled osteochondral defects, provided a 3D environment for cell adhesion and proliferation, and may recruit surrounding SMSCs migrate to the damage site and differentiate into cartilage, thereby effectively improving the repair of osteochondral defects.

## Figures and Tables

**Table 1:** Sample abbreviations used in this study.

Group	Composition
HSF	HAp+pure SF scaffold
HSP-1	HAp+SF with physical adsorption of PDGF
HSP-2	HAp+PDA-modified SF with no PDGF
HSPP	HAp+PDA-modified SF loaded with PDGF

Note: the nomenclature with H corresponds to scaffolds that contain HAp bone phase.

## Data Availability

The datasets used in the current study are available from the corresponding author on reasonable request by email.
